# ﻿*Alliumheterophyllum* (Amaryllidaceae), a new species from Henan, China

**DOI:** 10.3897/phytokeys.190.77449

**Published:** 2022-02-18

**Authors:** Deng-Feng Xie, Rui-Yu Cheng, Megan Price, Jun-Pei Chen, Jia-Qing Lei, Yi-Yang Zhang, Xing-Jin He

**Affiliations:** 1 Key Laboratory of Bio-Resources and Eco-Environment of Ministry of Education, College of Life Sciences, Sichuan University, 610065, Chengdu, Sichuan, China Sichuan University Chengdu China; 2 Sichuan Key Laboratory of Conservation Biology on Endangered Wildlife, College of Life Sciences, Sichuan University, Chengdu, 610065 Sichuan, China Sichuan University Chengdu China

**Keywords:** *Allium*, chromosome number, morphology, new species, phylogenetic analysis

## Abstract

*Alliumheterophyllum* D.F.Xie & X.J.He, **sp. nov.** (Amaryllidaceae), is a new species from Henan, China and is described based on morphological and molecular evidence. It is morphologically most similar to *A.dumebuchum* in the rhomboid scape in cross-section. However, distinctive differences were detected in perianth color, leaf shape and cross-section, flowers’ density as well as flowering season. Similarly, the karyotype of *A.heterophyllum* is 2*n* = 2*x* = 16 and in *A.dumebuchum* is 2*n* = 4*x* = 32. Phylogenetic analysis based on nuclear ribosomal Internal Transcribed Spacers (ITS) and three cpDNA regions strongly supports that *A.heterophyllum* is a member of Allium section Rhizirideum and sister to the other species of this section (e.g. *A.senescens*, *A.spirale*, and *A.prostratum*). Currently, only one population and approximately 120 individuals were discovered; the development of scenic spots in this region may affect its growth and threaten this population. Therefore, this new species is preliminarily considered as Near Threatened (NT) according to criteria of the IUCN Red List.

## ﻿Introduction

*Allium* L. is one of the largest genera of Amaryllidaceae (Fritsch et al. 2010; Li et al. 2010), and includes more than 950 species, that are characterized by rhizomatous or bulbous geophytes and widely used for food, medicine or as ornamental plants (e.g. garlic, leek, onion, and shallot) (Herden et al. 2016; Pellicer et al. 2017). Phylogenetic studies suggest that the genus *Allium* differentiated into three evolutionary lineages (Fritsch and Friesen 2002), and can be classified into 15 subgenera and 72 sections (Friesen et al. 2006). *Allium* species are widely distributed in the Northern Hemisphere, mostly from the dry subtropics to boreal zones, and the genus has two probable diversity centers, one stretching from the Mediterranean Basin to Central Asia and Pakistan, the other is in western North America (Fritsch and Friesen 2002). This genus is highly speciose in China with more than 150 taxa recorded and new species are frequently being discovered, such as *A.tetraploideum*, *A.xinlongense*, and *A.yingshanense* (Li et al. 2019; Xie et al. 2020a; Huang et al. 2021).

The typical section of AlliumsubgenusRhizirideum (G.Don ex W.D.J.Koch) Wendelbo, section Rhizirideum G.Don ex W.D.J.Koch has 25 species (including the recently published new species *A.dumebuchum*) (Jang et al. 2021), and their species are characterized by membranous tunics in enclosed bulbs, which are attached to horizontal rhizomes, hemicylindrical to plain leaf, and white to purple flowers (Sinitsyna et al. 2016). Additionally, the species of section Rhizirideum share a similar karyotype with the basic chromosome number of *x* = 8 (Sinitsyna et al. 2016). Most previous studies suggest that this section belongs to the third evolutionary lineage of *Allium* and is mainly distributed from Europe to East Asia, especially in temperate Asia (Fritsch and Friesen 2002; Friesen et al. 2006; Li et al. 2010; Choi et al. 2012; Sinitsyna et al. 2016). In China, species of this section are mainly distributed in Northern provinces, such as Inner Mongolia, Henan, and Xinjiang.

Previous phylogenetic studies suggested that the section Rhizirideum is a strong monophyletic unit (Friesen et al. 2006; Li et al. 2010). Following this, the nomenclature, distribution regions, and characteristics of all species in this section were further identified and listed (Sinitsyna et al. 2016; Sinitsyna and Friesen 2018). However, given the morphological diversity and prevalent polyploidy of this section (di-, tetra-, penta-, and hexaploids were detected) (Friesen 1988, 1992; Kamelin 2004), as well as the frequent discovery of new species, further studies are required to clarify taxonomic uncertainties.

**Figure 1.**
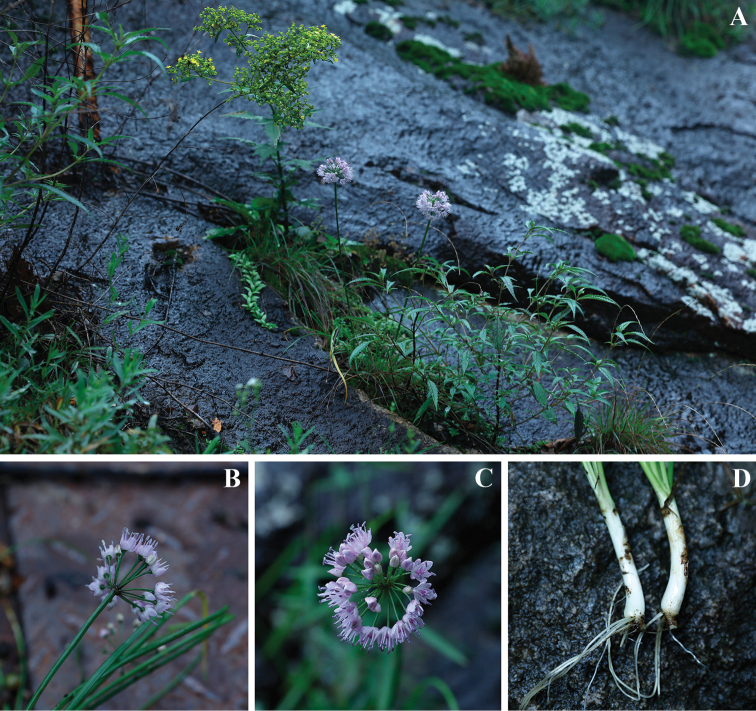
Living images of *Alliumheterophyllum***A** habitat, growing on the open slope of rock **B, C** inflorescence, light purple with loose flowers **D** bulb with the horizontal rhizome.

Many new *Allium* species have been described this year (Armağan 2021; Balos et al. 2021; Friesen et al. 2021; Pandey et al. 2021). During our field investigation in Songxian county in September 2021 (Henan province, China), we discovered a new *Allium* species (Fig. 1) that was similar to the members of Allium section Rhizirideum but had morphological differences. Thus, we conducted two field trips to collect fresh materials for further study. Here, we aimed to (1) investigate this new species *Alliumheterophyllum* based on morphological, karyotypic data and molecular approaches, and (2) conduct a comprehensive description of this new species, and thereby confirm the taxonomic relationships with other morphologically similar species in section Rhizirideum.

## ﻿Materials and methods

### ﻿Sampling and morphological analyses

Living plants and samples of *Alliumheterophyllum* were collected in Songxian county (33°41'25.61"N, 111°59'24.31"E, Altitude: 1347 m), Henan province, China. Voucher specimens were deposited at the herbarium of Sichuan University (SZ). Measurements of flowers, leaves, scapes, bulbs and rhizomes were taken from living plants and examined and measured by stereo binocular microscope (Nikon, Japan). A total of 18 diagnostic characteristics of the new species were identified and compared to six closely-related species in the Alliumsect.Rhizirideum.

### ﻿Karyotype analysis

Root tips were excised from the bulbs and pre-treated in saturated p-dichlorobenzene at 4 °C for 9 hours in the absence of light, then rinsed twice using distilled water and transferred to 3:1 ethanol-acetic acid for 10 hours. Subsequently, we rinsed the samples twice with distilled water and hydrolyzed in 1 mol/L HCL at 60 °C for 10 min. Finally, the samples were stained with the improved carbolfuchsin for one hour and squashed for observation. More than ten individuals were checked with three to five plates being investigated for each individual and well-spread metaphase plates were observed and further photographed using the Olympus BX43 electron microscope (Tokyo, Japan).

### ﻿DNA extraction, amplification and sequencing

Total DNA was extracted from silica gel dried young leaves of the new species using the Tiangen plant genomic DNA extraction kit (Tiangen Biotech, Beijing) according to the protocols of the manufacturer. The complete nucleotide ribosomal ITS region (ITS1, 5.8S and ITS2) was amplified using the ITS primers from White et al. (1990), and three other chloroplast regions (*ndhJ*-*trnF*, *psbD*-*trnT* and *psbJ*-*petA*) were also collected based on corresponding primers (Taberlet et al. 1991; Shaw et al. 2007). The detailed primers and amplification information are shown in Table 1. All PCR products were visualized on 2% agarose TAE gel and sent to Sangon Biotech Institute (Shanghai, China) for sequencing. The DNA sequences generated in this study have been deposited in NCBI (Suppl. material 1: Table S1).

**Table 1.** Primers and amplification information were used for DNA barcoding in this study.

Fragment	Marker	Sequence 5'–3'	Tm (°C)	Reference
*ITS*	*ITS4*	TCCTCCGCTTATTGATATGC	55.0	White et al. 1990
	*ITS5*	GGAAGTAAA AGTCGTAACAAGG		
*ndhJ-trnF*	*ndhJ*	ATGCCYGAAAGTTGGATAGG	54.2	Shaw et al. (2007)
	*tabE*	GGTTCAAGTCCCTCTATCCC		Taberlet et al. (1991)
*psbD-trnT*	*psbD*	CTCCGTARCCAGTCATCCATA	54.8	Shaw et al. (2007)
	*trnT^GGU^*	CCCTTTTAACTCAGTGGTAG		
*psbJ-petA*	*psbJ*	ATAGGTACTGTARCYGGTATT	54.5	Shaw et al. (2007)
	*petA*	AACARTTYGARAAGGTTCAATT		

The PCR program began with 4-min initial denaturing at 94 °C followed by 35 cycles of 1-min denaturation at 94 °C, 1-min annealing at abovementioned Tm, and 1.5-min extension at 72 °C, a final extension was run for 5 min at 72 °C.

### ﻿Sequence download, extraction and phylogenetic analysis

We downloaded an extensive dataset of ITS, cpDNA regions and chloroplast genomes from NCBI to better perform the phylogenetic analysis and confirm the systematic position of this new species. We downloaded 107 ITS sequences from 43 *Allium* species, and 69 cpDNA regions and 55 chloroplast genomes from 10 and 37 *Allium* species, respectively. To conduct the phylogenetic analysis using the three cpDNA regions, we extracted each cpDNA region from the 56 chloroplast genomes. The detailed Genbank accession information of all sequences is provided in Suppl. material 1: Table S1.

Newly sequenced ITS and cpDNA regions were assembled using the SeqMan software (Burland 2000), and then aligned with Clustal X (Jeanmougin et al. 1998) and further manually adjusted in MEGA 7.0 (Kumar et al. 2016). Two methods (Maximum likelihood and Bayesian inference) were used to perform the phylogenetic analysis. Maximum likelihood (ML) analyses were conducted in RAxML 8.2.8 (Stamatakis 2014) with GTR+G model and 1,000 bootstrap replicates. Bayesian inference (BI) was performed in MrBayes v 3.2.7 (Ronquist et al. 2012) with GTR+G being selected as the optimal model of nucleotide substitution using the Akaike information criterion (AIC; Burnham and Anderson 2002) as implemented in IQ-TREE (Trifinopoulos et al. 2016). The Markov Chains (including three heated chains and one cold chain) were run for 1 × 10^8^ generations with a sample frequency of 50 and the initial 20% of the samples discarded as burn-in to confirm the stationarity. The remaining trees were used to build a 50% majority-rule consensus tree.

## ﻿Results and discussion

### ﻿Taxonomy treatment

#### ﻿ Allium heterophyllum

Taxon classificationPlantaeAsparagalesAmaryllidaceae

D.F.Xie & X.J.He sp. nov.

91F89E04-C877-5143-B786-DAA9CEE9618D

urn:lsid:ipni.org:names:77255008-1

Figs 1 , 2 , 3

##### Type.

China. Henan Province: Songxian County, Longchiman mountains, 111°59'24.31"E, 33°41'25.61"N, 1347 m alt., 04 September 2021, Anonymous, XDF20210904 (Holotype: SZ; Isotype: SZ).

##### Diagnosis.

**Figure 2.**
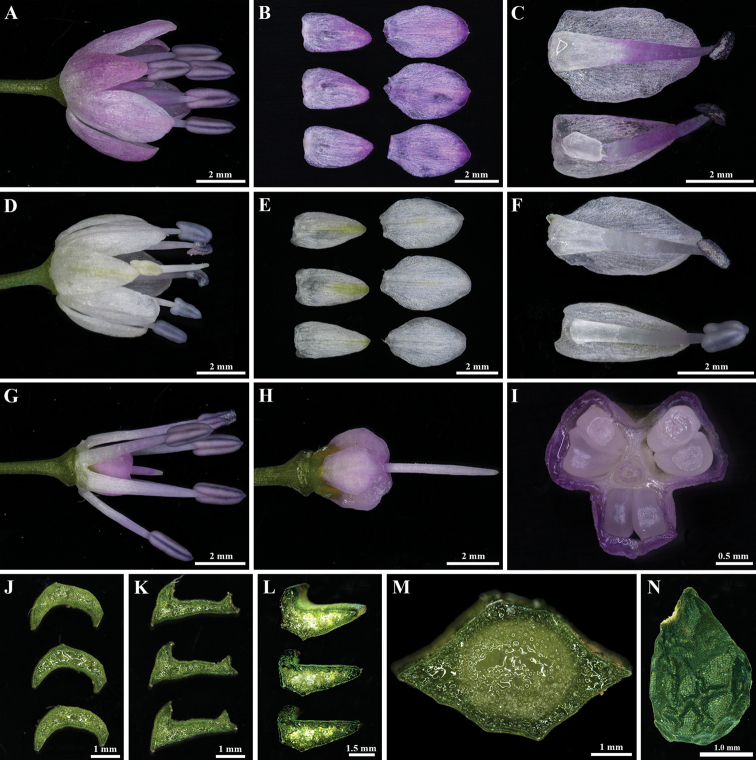
Morphological characters of *Alliumheterophyllum***A, D** single flower with light purple or white color **B, E** outer (left) and inner (right) tepals **C, F** inner (top) and outer (bottom) tepals and stamen **G** stamen and trait at the base **H** ovary **I** cross-section of ovary showing the carpels **J–L** the cross-section of leaf showing the blade characters **M** cross-section of rhomboid scapes **N** seeds’ characters.

*Alliumheterophyllum* resembles *A.dumebuchum* due to its rhomboid scape in cross-section. However, it is clearly distinguished from *A.dumebuchum* in perianth (white to light purple vs. light purple), leaves (not tortuous and not flesh vs. slight tortuous and flesh), the cross-section of leaves (two types vs. one type), flowers’ density (loose inflorescence vs. many-flowered) (Fig. 2; Table 2), flowering season (late August to September vs. late September to October), and karyotype (2n = 16 vs. 2n = 32) (Fig. 4). Compared to other *Rhizirideum* species (e.g., *A.scenescens*, *A.spirale* and *A.spurium*), *A.heterophyllum* also shows distinctive morphological characters, such as rhomboid scape in cross-section, unique two types of leaves, loose flowers, white to light purple color of perianth and filaments, and flowering season.

**Table 2.** The diagnostic morphological characters of *Alliumheterophyllum* and related species.

Character		*A.heterophyllum*	*A.dumebuchum*	*A.spirale*	*A.spurium*	*A.minus*	*A.senescens*	*A.nutans*
**Bulb**	growth pattern	solitary, paired or clustered	clustered	clustered	solitary or paired	clustered	solitary or paired	solitary or paired
	shape	conical to ovate-cylindric	conical to cylindric	conical to cylindric	cylindric to conical-cylindric	conical to cylindric	conical to ovate-cylindric	narrowly cylindric to subconical
	diameter (mm)	5.0–15.0	9.6–15.0	5.0–15.0	5.0–15.0	4.3–8.6	10.0–20.0	15.0–20.0
**Rhizome**	growth pattern	oblique to horizontal	oblique to horizontal	horizontal	horizontal	oblique	horizontal	horizontal or oblique and stout
**Leaf sheath**	exposed or buried	exposed	exposed	buried	buried	exposed	exposed	exposed
**Leaf blade**	shape	linear, solid, not fleshy, canaliculated with one bulge in the back or flat with irregularly one or two-edged margin	ascending, slightly tortuous, linear, flat and solid in cross-section, flesh, apex obtuse to rounded	linear, spirally tortuous, flat, main veins and margins minutely scabrous-denticulate, rarely smooth, fleshy, apex obtuse	narrowly linear, straight, flat to convex-flat, fleshy, margin minutely scabrous, apex acute to gradually attenuate, truncate	ascending, spirally tortuous, flat, fleshy, linear, solid, fleshy, obtuse to rounded at apex	spirally arranged, broadly linear, fleshy, sometimes slightly falcate	broadly linear, subfalcate, flat, thick, fleshy, smooth, apex obtuse
	length (cm)	15–45.0	19.5–38.0	20.0–45.0	15–30.0	11.4–24.5	23.0–45.0	30.0–55.0
	width (mm)	1.5–4.0	3.8–13.0	4.0–10.0	1.5–4.0	2.8–4.5	5.0–15.0	6.0–15.0
**Umbel**	shape	hemispheric, loose	subglobose, many-flowered	hemispheric to subglobose, many-flowered.	laxly hemispheric, many-flowered.	hemispheric	hemispheric to globose, many-flowered	globose, densely many-flowered
**Scape**	cross-section	rhomboid	rhomboid	flattened-winged	rhomboid to subterete	subterete	subterete	2-angled, narrowly 2-winged
	length (cm)	25.0–45.0	23.4–49.0	33.0–65.0	10.0–40.0	11.7–20.5	25.8–70.0	30.0–60.0
	diameter (mm)	1.5–2.5	2.5–5.6	4.0–5.1	1.5–2.5	1.5–1.6	3.0–5.5	3.5–6.0
**Pedicel**	length (mm)	10.0–15.0	9.8–11.2	6.0–12.4	7.6–11.1	8.7–11.1	8.0–13.0	9.0–15.5
**Spathe**		1-valved, persistent and inconspicuous	unknown	2-valved, persistent	2-valved, usually caducous	unknown	2-valved, persistent	2-valved, persistent
**Perianth**	color	white to light purple	light purple	reddish purple	strong purple or pale purple	pale purple	pale purple	pale red to pale purple
**Inner tepal**	shape	elliptical	elliptical to ovate-elliptical	ovate-elliptical	ovate-elliptical	elliptical	elliptical	ovate
	length (mm)	4.0–6.0	5.2–7.2	4.0–6.8	3.9–6.3	4.0–4.8	4.3–6.4	5.0–6.5
	width (mm)	2.2–2.5	3.4–4.5	2.0–4.2	2.2–3.4	1.2–1.9	1.8–2.9	2.2–3.0
**Outer tepal**	shape	ovate-elliptical	ovate-elliptical	ovate-elliptical	ovate-elliptical	ovate-oblong	ovate-elliptical	narrowly ovate
	length (mm)	3.0–4.0	4.8–6.1	3.1–5.0	2.9–5.2	3.7–4.6	3.1–5.2	4.5–5.5
	width (mm)	1.6–1.9	2.1–3.7	1.3–3.0	1.1–2.3	1.1–1.7	1.1–2.5	1.5–2.0
**Filament**	exsertion	exserted	exserted	exserted	exserted	included	exserted	exserted
	length (mm)	6.3–7.5	6.2–8.4	5.3–8.8	5.0–7.0	3.2–4.4	4.6–6.9	6.5–8.5
**Base of inner filament**	shape	narrowly triangular	narrowly triangular	subulate	subulate	broadened in the lower half	broadened in the lower half	broadened in the lower half, 1-toothed on each side
**Anther**	color	purple grey	purple	purple	yellow	reddish	black or yellowish-brown	yellow
	length (mm)	1.8–2.3	2.2–2.5	1.7–2.2	1.7–2.0	1.3–1.4	1.5–2.0	1.8–2.3
	width (mm)	0.9–1.4	0.9–1.1	0.7–1.0	0.6–0.8	0.6–0.8	0.5–0.8	0.6–0.9
**Ovary**	shape	obovoid	obovoid	broadly ovoid	ovoid	obovoid	obovoid	oblong-globose
**Flowering season**		late Aug. to Sep.	late Sep. to Oct.	Aug. to Sep.	Jul. to Aug.	May to Jul.	Jul. to Aug.	Jun. to Aug.
**Chromosome number (2n)**		2n = 16	2n = 32	2n = 16, 32	2n = 16, 32	2n = 16	2n = 32	2n = 16, 17, 24, 28, 32, 44, 48, 56, 64, 72

**Figure 3.**
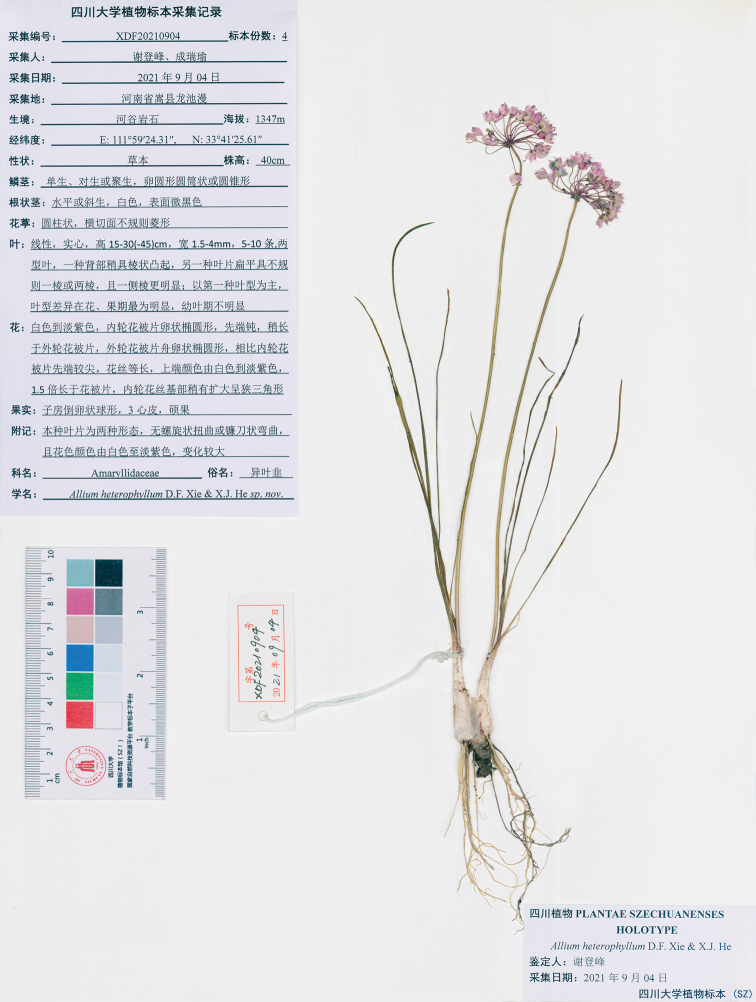
Photograph of the Holotype of *Alliumheterophyllum*.

**Figure 4.**
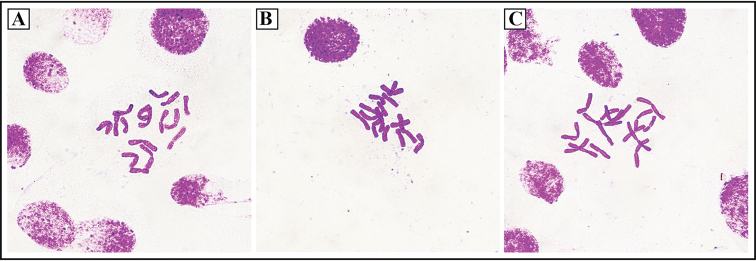
The chromosome complement of *Alliumheterophyllum* (2n = 2*x* = 16).

##### Description.

Perennial herbs, bulbs solitary, paired or clustered, ovate-cylindric or conical, 5.0–15 mm in diameter, tunics membranous, white, attached to a horizontal or oblique rhizome, 5.0–20.0 mm in diameter, surface usually blackish gray. Leaves linear, 5.0–10.0, solid in cross-section, 1.0–30.0 (–45.0) cm long and 1.5–4.0 mm wide, usually shorter than scape, sometimes equal to the length of scape, exposed sheaths in 1/7; cross-section of leaves exposed two types of morphologies, canaliculated with one bulge in the back or flat with irregularly one or two-edged margin; the leaves’ shape differences are most obvious in flower and fruit periods but not obvious in young leaves. Scapes rhomboid, solid in cross-section, 25.0–45.0 cm long, and 15.0–25.0 mm in diameter. Spathe 1-valved, persistent and inconspicuous; inflorescence umbellate hemispheric, loose. Pedicels equal, 10.0–15.0 mm; perianth white to light purple, inner tepals 4.0–6.0 mm, longer than outer ones, elliptical, apex obtuse; outer tepals 3.0–4.0 mm, ovate-elliptical. Filaments equal and exserted, white to light purple in the upper part, 1.5 × as long as perianth segments and connate at the base of the perianth. Outer one subulate, inner filaments narrowly triangular; anthers elliptical, purple-grey. Ovary obovoid, trigonous, white to light purple, without concave nectaries. 3 carpel and ovules 2 per locule, style exserted, stigma punctiform. Capsule obovate; seeds black, rhomboidal, 1.5–2.0 mm wide and 2.5–3.0 mm long (Fig. 2; Table 2).

##### Etymology.

The new species epithet “*heterophyllum*” is based on the unique leaves’ characters, its leaves exposed two types of morphologies, canaliculated with one bulge in the back or flat with irregularly one or two-edged margin, and the differences in the leaves are most obvious in flowering and fruit periods. (Fig. 2).

##### Phenology.

Through two field investigations, *A.heterophyllum* was flowering from late August to September and fruiting from late September to October.

##### Habitat and ecology.

Currently, *A.heterophyllum* is only known from the type population in Longchiman Mountains in Songxian County, Henan, China. This species grows on the open slope of rock by the river with a small amount of soil attached, sometimes rooting in crevices, holes or steps of the rock face at an elevation from 1250 m to 1400 m.

##### Chinese name.

Yi Ye Jiu (异叶韭).

### ﻿Morphological analysis

The latest study suggested that as many as one-third of the species could face extinction within the next 50 years due to biodiversity loss resulting from climate change (Román-Palacios and Wiens 2020). Therefore, the discovery of a new species is always awe-inspiring news; in 2020, more than 300 new species were discovered in China (Du et al. 2021), which fills us with hope. In this study, we confirmed a new *Allium* species named *Alliumheterophyllum* based on morphological comparisons and molecular analysis. This new species is morphologically most similar to *A.dumebuchum*, which is endemic to Ulleungdo island of Korea (Jang et al. 2021). Although these two species shared similar rhomboid scape in cross-section, their flowering times are different (late August to September vs. late September to October), and distinctive morphological characters were also detected in the perianth color, leaves’ shape and cross-section, and flowers’ density (Table 2). Additionally, *A.heterophyllum* is a diploid species with a somatic chromosome number of 2n = 16, while *A.dumebuchum* is a tetraploid species with 2n = 32 (Fig. 4). Through analyzing 18 diagnostic characteristics, we also found obvious differences between *A.heterophyllum* and other species in section Rhizirideum (e.g., *A.senescens*, *A.prostratum*, and *A.spirale*), such as the leaves, which in *A.dumebuchum*, *A.minus* and *A.senescens* are fleshy and glaucous and leathery and lustrous in *A.spirale* and *A.spurium*. Moreover, the flowering season is also different, because other species of section Rhizirideum usually bloom from May or July, except *A.spirale*, which blooms from August to September. Further differences are also reflected in the color of perianths, filaments and anthers (Table 2).

### ﻿Phylogenetic analysis

**Figure 5.**
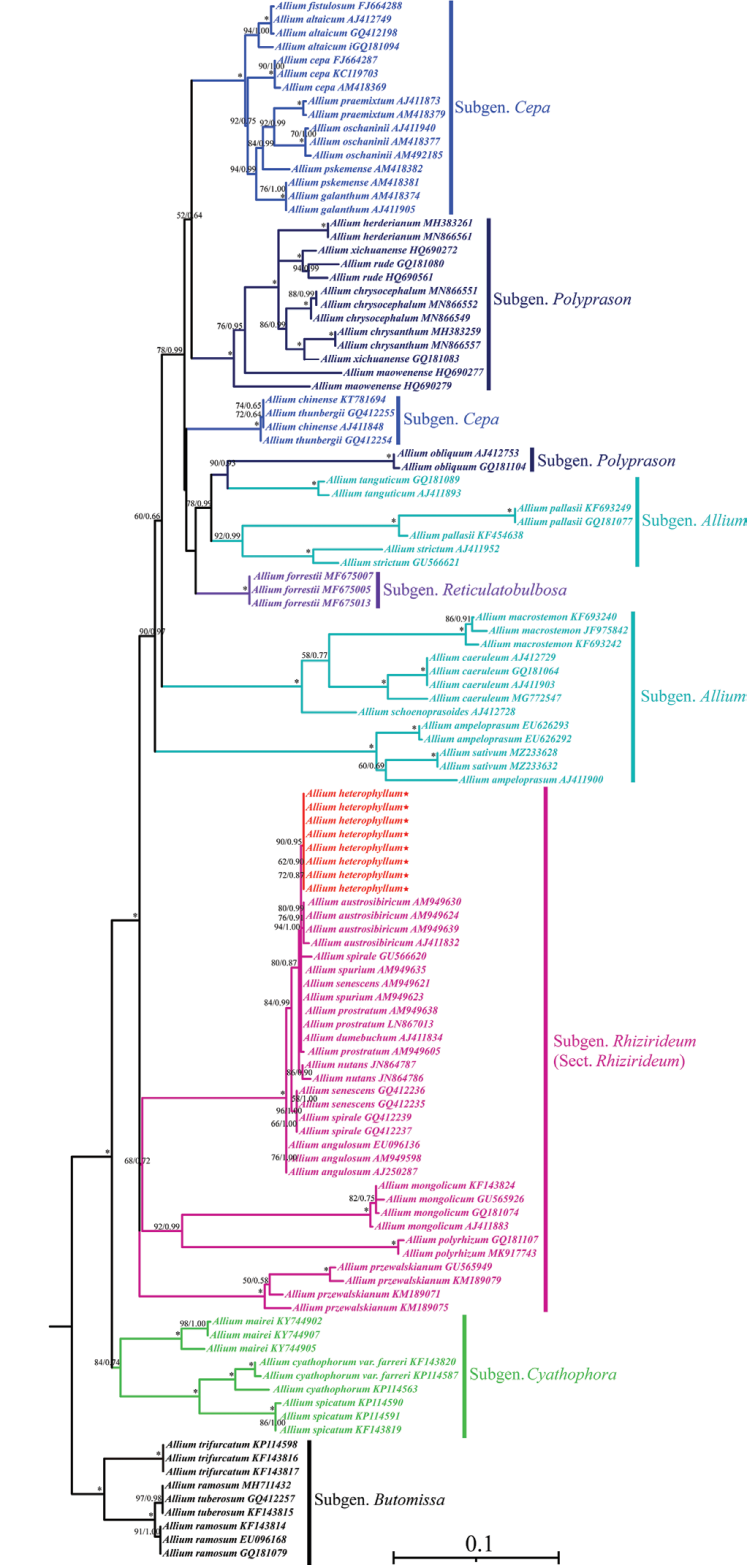
Phylogenetic relationships inferred from ITS. Trees constructed with maximum likelihood (ML) and Bayesian inference (BI). Support values reported above the branches are bootstrap values of ML and posterior probability of BI. * = maximum support in the two analyses. Samples of Alliumsect.Rhizirideum are in rose red and the sequences of *Alliumheterophyllum* are in red and markered with the star.

**Figure 6.**
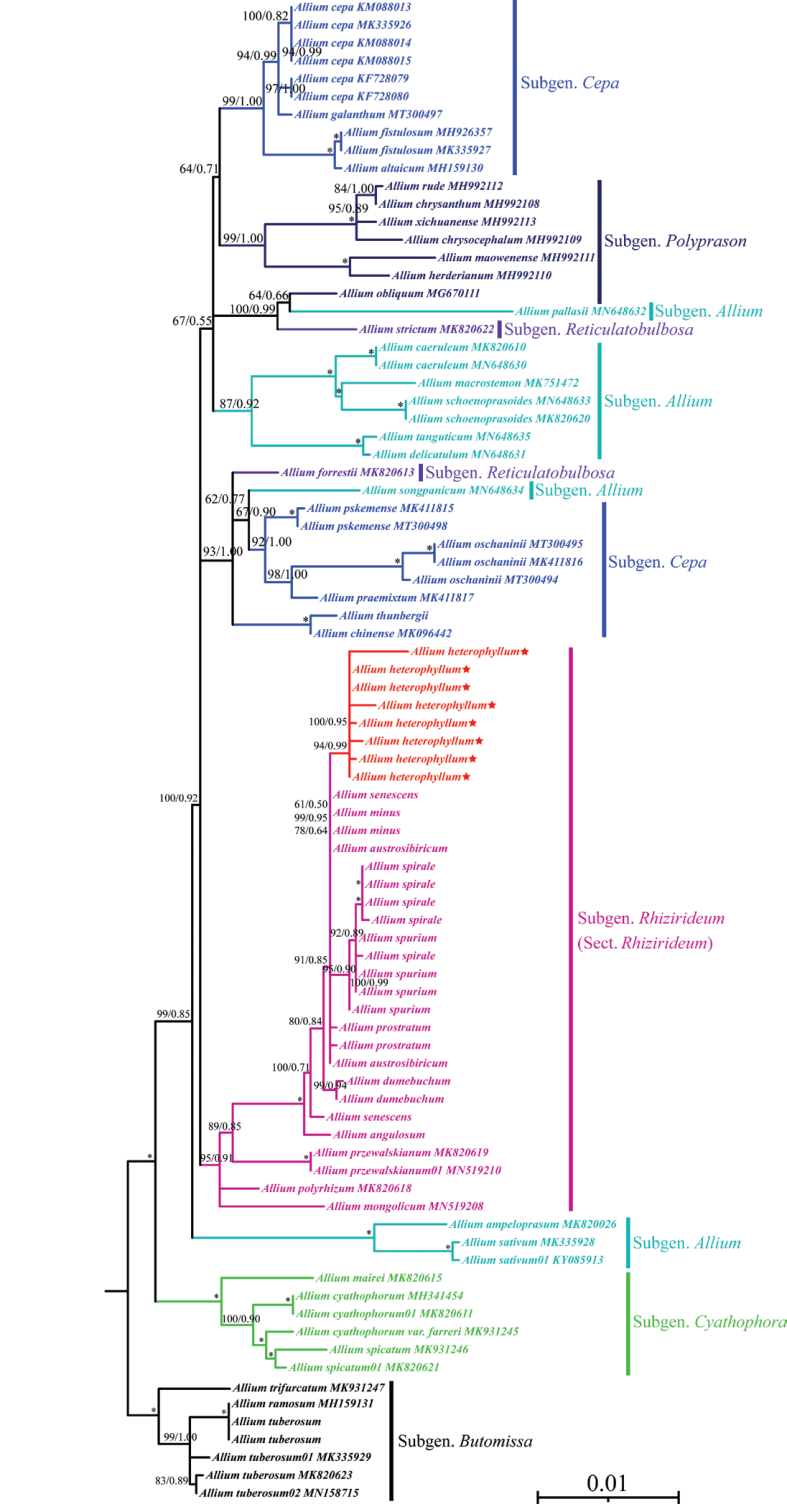
Phylogenetic relationships inferred from three cpDNA alignments. Trees constructed with maximum likelihood (ML) and Bayesian inference (BI). Support values reported above the branches are bootstrap values of ML and posterior probability of BI. * = maximum support in the two analyses. Branches of Alliumsect.Rhizirideum are in rose red and the samples of *Alliumheterophyllum* are in red and markered with the star.

Total ITS alignments were 703 bp in length with 446 variable sites (63.44%) and 421 parsimony-informative characters (PICs; 59.89%). Alignments of the three cpDNA regions possessed 3708 bp with 707 variable sites (19.07%) and 432 PICs (11.65%). The phylogenetic tree from ITS data was consistent with the cpDNA data set tree, in which the subgenus Rhizirideum is monophyletic and subgenera *Allium*, *Cepa*, and *Polyprason* are polyphyletic (Figs 5, 6). Moreover, all individuals of *A.heterophyllum* clustered into monophyly in the ITS and cpDNA trees with high support values (Figs 5, 6).

Our phylogenetic results detected a similar topology to previous studies (Friesen et al. 2006; Li et al. 2010; Choi et al. 2012; Sinitsyna et al. 2016; Xie et al. 2020b; Jang et al. 2021). ITS and cpDNA regions strongly support that *A.heterophyllum* is a member of section Rhizirideum, and sister to the other species of section Rhizirideum (e.g. *A.senescens*, *A.spirale*, and *A.prostratum*). Although *A.heterophyllum* is morphologically most similar to *A.dumebuchum*, these two species are not closely related in the phylogenetic trees. According to previous studies, species in the section Rhizirideum are very widely distributed across the world and exhibit complicated relationships (Friesen et al. 2006; Choi et al. 2012; Sinitsyna et al. 2016; Jang et al. 2021), and this section is also regarded as a difficult taxon concerning classification and identification. Thus, morphological and phylogenetic analyses should be conducted at the population level in the future, thereby better investigating the species’ relationships.

### ﻿Conservation status

Through our field investigation, only one population with approximately 120 individuals of this species was discovered in the Longchiman Mountains. Given the development of tourism in this region, it is possible that this population may be threatened by pedestrian traffic, pollution, infrastructure development and other threatening processes associated with tourism. Therefore, this species is preliminarily considered as Near Threatened (NT) according to the IUCN Red List Categories and Criteria (IUCN 2019).

## Supplementary Material

XML Treatment for Allium heterophyllum

## Supplementary materials

Supplementary material 1 Table S1 Data type Sequence data accession NCBI numbers. Explanation note List of species accession NCBI numbers in this study. https://binary.pensoft.net/file/647504This dataset is made available under the Open Database License (http://opendatacommons.org/licenses/odbl/1.0/). The Open Database License (ODbL) is a license agreement intended to allow users to freely share, modify, and use this Dataset while maintaining this same freedom for others, provided that the original source and author(s) are credited.Deng-Feng Xie, Rui-Yu Cheng, Megan Price, Jun-Pei Chen, Jia-Qing Lei, Yi-Yang Zhang, Xing-Jin He
