# Supplementary material for: ﻿Alliumheterophyllum (Amaryllidaceae), a new species from Henan, China
Source: PhytoKeys. 2022 Feb 18;190:53–67. doi: 10.3897/phytokeys.190.77449 (PMC8881438; doi:10.3897/phytokeys.190.77449)
Supplement: Supplementary material 1 — Table S1 [file phytokeys-190-053-s001.docx]

**TableS1 List of species accession NCBI numbers in this study.**

| Species | *psbJ-petA* | *ndhJ-tabF* | *psbD-trnT* |
| --- | --- | --- | --- |
| *Allium angulosum* | MW478175 | MW478211 | MW478283 |
| *Allium austrosibiricum* | MW478174 | MW478210 | MW478282 |
|  | MW478173 | MW478209 | MW478281 |
| *Allium dumebuchum* | MW478172 | MW478208 | MW478280 |
|  | MW478171 | MW478207 | MW478279 |
| *Allium minus* | MW478170 | MW478206 | MW478278 |
|  | MW478169 | MW478205 | MW478277 |
| *Allium prostratum* | MW478168 | MW478204 | MW478276 |
|  | MW478167 | MW478203 | MW478275 |
| *Allium senescens* | MW478166 | MW478202 | MW478274 |
|  | MW478165 | MW478201 | MW478273 |
| *Allium spirale* | MW478155 | MW478191 | MW478263 |
|  | MW478159 | MW478195 | MW478267 |
|  | MW478158 | MW478194 | MW478266 |
|  | MW478151 | MW478187 | MW478259 |
|  | MW478150 | MW478186 | MW478258 |
| *Allium spurium* | MW478144 | MW478180 | MW478252 |
|  | MW478143 | MW478179 | MW478251 |
|  | MW478148 | MW478184 | MW478256 |
|  | MW478147 | MW478183 | MW478255 |
| *Allium thunbergii* | MW478142 | MW478178 | MW478250 |
| *Allium tuberosum* | MW478141 | MW478177 | MW478249 |
|  | MW478140 | MW478176 | MW478248 |
| ***Allium heterophyllum*** | **OK605995** | **OK605987** | **OK605979** |
| ***Allium heterophyllum*** | **OK605996** | **OK605988** | **OK605980** |
| ***Allium heterophyllum*** | **OK605997** | **OK605989** | **OK605981** |
| ***Allium heterophyllum*** | **OK605998** | **OK605990** | **OK605982** |
| ***Allium heterophyllum*** | **OK605999** | **OK605991** | **OK605983** |
| ***Allium heterophyllum*** | **OK606000** | **OK605992** | **OK605984** |
| ***Allium heterophyllum*** | **OK606001** | **OK605993** | **OK605985** |
| ***Allium heterophyllum*** | **OK606002** | **OK605994** | **OK605986** |

The chloroplast genomes downloaded from NCBI.

| Species | Chloroplast genome accession numbers | | | | |
| --- | --- | --- | --- | --- | --- |
| *Allium cepa* | KF728079 | KM088014 | KM088013 | MK335926 | KM088015 |
| *Allium galanthum* | MT300498 | MT300497 |  |  |  |
| *Allium fistulosum* | MH926357 | MK335927 |  |  |  |
| *Allium altaicum* | MH159130 |  |  |  |  |
| *Allium rude* | MH992112 |  |  |  |  |
| *Allium xichuanense* | MH992113 |  |  |  |  |
| *Allium chrysanthum* | MH992108 |  |  |  |  |
| *Allium chrysocephalum* | MH992109 |  |  |  |  |
| *Allium maowenense* | MH992111 |  |  |  |  |
| *Allium herderianum* | MH992110 |  |  |  |  |
| *Allium caeruleum* | MK820610 | MN648630 |  |  |  |
| *Allium macrostemon* | MK751472 |  |  |  |  |
| *Allium schoenoprasoides* | MK820620 | MN648633 |  |  |  |
| *Allium delicatulum* | MN648631 |  |  |  |  |
| *Allium tanguticum* | MN648635 |  |  |  |  |
| *Allium teretifolium* | MN648632 |  |  |  |  |
| *Allium obliquum* | MG670111 |  |  |  |  |
| *Allium strictum* | MK820622 |  |  |  |  |
| *Allium pallasii* | MN648632 |  |  |  |  |
| *Allium oschaninii* | MK411816 | MT300494 | MT300495 |  |  |
| *Allium praemixtum* | MK411817 |  |  |  |  |
| *Allium pskemense* | MK411815 | MT300498 |  |  |  |
| *Allium forrestii* | MK820613 |  |  |  |  |
| *Allium songpanicum* | MN648634 |  |  |  |  |
| *Allium chinense* | MK096442 |  |  |  |  |
| *Allium przewalskianum* | MK820619 | MN519210 |  |  |  |
| *Allium polyrhzum* | MK820618 |  |  |  |  |
| *Allium mongolicum* | MN519208 |  |  |  |  |
| *Allium sativum* | KX683282 | MK335928 | KY085913 |  |  |
| *Allium ampeloprasum* | MK820026 |  |  |  |  |
| *Allium spicatum* | MK931246 | MK820621 |  |  |  |
| *Allium cyathophorum var. farreri* | MK931245 |  |  |  |  |
| *Allium cyathophorum* | MH341454 | MK820611 |  |  |  |
| *Allium mairei* | MK820615 |  |  |  |  |
| *Allium trifurcatum* | MK931247 |  |  |  |  |
| *Allium ramosum* | MH159131 |  |  |  |  |
| *Allium tuberosum* | MK335929 | MN158715 | MK820623 |  |  |

The all ITS sequences used in this study.

| Species | NCBI accession number |
| --- | --- |
| *Allium altaicum* | AJ412749 |
| *Allium altaicum* | GQ412198 |
| *Allium altaicum* | GQ181094 |
| *Allium ampeloprasum* | AJ411900 |
| *Allium ampeloprasum* | EU626292 |
| *Allium ampeloprasum* | EU626293 |
| *Allium angulosum* | AJ250287 |
| *Allium angulosum* | AM949598 |
| *Allium angulosum* | EU096136 |
| *Allium austrosibiricum* | AM949624 |
| *Allium austrosibiricum* | AM949630 |
| *Allium austrosibiricum* | AM949639 |
| *Allium caeruleum* | AJ411903 |
| *Allium caeruleum* | AJ412729 |
| *Allium caeruleum* | GQ181064 |
| *Allium caeruleum* | MG772547 |
| *Allium cepa* | AM418369 |
| *Allium cepa* | FJ664287 |
| *Allium cepa* | KC119703 |
| *Allium chinense* | AJ411848 |
| *Allium chinense* | KT781694 |
| *Allium chrysanthum* | MH383259 |
| *Allium chrysanthum* | MN866557 |
| *Allium chrysocephalum* | MN866549 |
| *Allium chrysocephalum* | MN866551 |
| *Allium chrysocephalum* | MN866552 |
| *Allium cyathophorum* | KF143820 |
| *Allium cyathophorum* | KP114587 |
| *Allium cyathophorum* | KP114563 |
| *Allium fistulosum* | FJ664288 |
| *Allium forrestii* | MF675005 |
| *Allium forrestii* | MF675007 |
| *Allium forrestii* | MF675013 |
| *Allium galanthum* | AJ411905 |
| *Allium galanthum* | AM418374 |
| *Allium herderianum* | MH383261 |
| *Allium herderianum* | MN866561 |
| *Allium macrostemon* | JF975842 |
| *Allium macrostemon* | KF693240 |
| *Allium macrostemon* | KF693242 |
| *Allium mairei* | KY744902 |
| *Allium mairei* | KY744907 |
| *Allium mairei* | KY744905 |
| *Allium maowenense* | HQ690279 |
| *Allium maowenense* | HQ690277 |
| *Allium mongolicum* | AJ411883 |
| *Allium mongolicum* | GQ181074 |
| *Allium mongolicum* | KF143824 |
| *Allium mongolicum* | GU565926 |
| *Allium nutans* | JN864786 |
| *Allium nutans* | JN864787 |
| *Allium austrosibiricum* | AJ411832 |
| *Allium obliquum* | AJ412753 |
| *Allium obliquum* | GQ181104 |
| *Allium oschaninii* | AJ411940 |
| *Allium oschaninii* | AM418377 |
| *Allium oschaninii* | AM492185 |
| *Allium pallasii* | GQ181077 |
| *Allium pallasii* | KF693249 |
| *Allium pallasii* | KF454638 |
| *Allium polyrhizum* | GQ181107 |
| *Allium polyrhizum* | MK917743 |
| *Allium praemixtum* | AJ411873 |
| *Allium praemixtum* | AM418379 |
| *Allium prostratum* | AM949638 |
| *Allium prostratum* | AM949605 |
| *Allium przewalskianum* | GU565949 |
| *Allium przewalskianum* | KM189079 |
| *Allium przewalskianum* | KM189071 |
| *Allium przewalskianum* | KM189075 |
| *Allium pskemense* | AM418381 |
| *Allium pskemense* | AM418382 |
| *Allium ramosum* | EU096168 |
| *Allium ramosum* | GQ181079 |
| *Allium ramosum* | KF143814 |
| *Allium ramosum* | MH711432 |
| *Allium rude* | GQ181080 |
| *Allium rude* | HQ690561 |
| *Allium sativum* | MZ233628 |
| *Allium sativum* | MZ233632 |
| *Allium schoenoprasoides* | AJ412728 |
| *Allium dumebuchum* | AJ411834 |
| *Allium senescens* | AM949621 |
| *Allium senescens* | GQ412235 |
| *Allium senescens* | GQ412236 |
| *Allium spicatum* | KF143819 |
| *Allium spicatum* | KP114590 |
| *Allium spicatum* | KP114591 |
| *Allium spirale* | GU566620 |
| *Allium spirale* | GQ412237 |
| *Allium spirale* | GQ412239 |
| *Allium spurium* | AM949623 |
| *Allium spurium* | AM949635 |
| *Allium strictum* | AJ411952 |
| *Allium strictum* | GU566621 |
| *Allium tanguticum* | AJ411893 |
| *Allium tanguticum* | GQ181089 |
| *Allium prostratum* | LN867013 |
| *Allium thunbergii* | GQ412254 |
| *Allium thunbergii* | GQ412255 |
| *Allium trifurcatum* | KF143816 |
| *Allium trifurcatum* | KP114598 |
| *Allium trifurcatum* | KF143817 |
| *Allium tuberosum* | GQ412257 |
| *Allium tuberosum* | KF143815 |
| *Allium xichuanense* | HQ690272 |
| *Allium xichuanense* | GQ181083 |
| ***Allium heterophyllum*** | **OK572470** |
| ***Allium heterophyllum*** | **OK572471** |
| ***Allium heterophyllum*** | **OK572472** |
| ***Allium heterophyllum*** | **OK572473** |
| ***Allium heterophyllum*** | **OK572474** |
| ***Allium heterophyllum*** | **OK572475** |
| ***Allium heterophyllum*** | **OK572476** |
| ***Allium heterophyllum*** | **OK572477** |
